# Heart rate reveals torpor at high body temperatures in lowland tropical free-tailed bats

**DOI:** 10.1098/rsos.171359

**Published:** 2017-12-20

**Authors:** M. Teague O'Mara, Sebastian Rikker, Martin Wikelski, Andries Ter Maat, Henry S. Pollock, Dina K. N. Dechmann

**Affiliations:** 1 Department of Migration and Immuno-Ecology, Max Planck Institute for Ornithology, Radolfzell, Germany; 2 Department of Biology, University of Konstanz, Konstanz, Germany; 3 Department of Chemistry, University of Konstanz, Konstanz, Germany; 4 Smithsonian Tropical Research Institute, Balboa, Ancón, Panama City, Panamá; 5 Department of Behavioural Neurobiology, Max Planck Institute for Ornithology, Seewiesen, Germany; 6 Program in Ecology, Evolution and Conservation Biology, University of Illinois at Urbana-Champaign, Urbana, IL, USA; 7 Department of Wildlife, Fish and Conservation Biology, Colorado State University, Fort Collins, CO, USA

**Keywords:** heterothermy, endothermy, body temperature, energetics, flight, tropics

## Abstract

Reduction in metabolic rate and body temperature is a common strategy for small endotherms to save energy. The daily reduction in metabolic rate and heterothermy, or torpor, is particularly pronounced in regions with a large variation in daily ambient temperature. This applies most strongly in temperate bat species (order Chiroptera), but it is less clear how tropical bats save energy if ambient temperatures remain high. However, many subtropical and tropical species use some daily heterothermy on cool days. We recorded the heart rate and the body temperature of free-ranging Pallas' mastiff bats (*Molossus molossus*) in Gamboa, Panamá, and showed that these individuals have low field metabolic rates across a wide range of body temperatures that conform to high ambient temperature. Importantly, low metabolic rates in controlled respirometry trials were best predicted by heart rate, and not body temperature*. Molossus molossus* enter torpor-like states characterized by low metabolic rate and heart rates at body temperatures of 32°C, and thermoconform across a range of temperatures. Flexible metabolic strategies may be far more common in tropical endotherms than currently known.

## 1. Introduction

Maintaining body temperature (*T*_b_) is energetically costly, particularly when ambient temperatures (*T*_a_) are cold and food resources scarce [1]. This problem would appear to be minimal in the tropics, where *T*_a_ is generally high. However, there can be strong resource ephemerality in tropical systems, making energy conservation a priority [2–4]. As seasonality becomes more intense with climate change [5], understanding individual-level physiological mechanisms and energetic limitations will be essential to understand population-level adaptation [6]. This is particularly true for tropical species, which may be limited in their energetic flexibility due to current adaptation to high *T*_a_ [7–11].

One physiological strategy to minimize energetic expenditure is the controlled reduction of the metabolic rate (MR) and a subsequent reduction in body temperature (*T*_b_). Often described as daily heterothermy or torpor [1,12,13], this phenomenon is widespread among mammals and birds, and is typically perceived as a response to mitigate low food availability and low *T*_a_ [13]. It is particularly pronounced in the large and diverse order of bats (Chiroptera), which need effective energy-saving strategies due to their small size, high metabolic requirements, and loss of large amounts of heat and water through large, naked wing membranes [14]. Although most common in temperate regions, subtropical and tropical species of bats also enter torpor and many species exhibit heterothermy when *T*_a_ falls below a threshold (e.g. 24°C: [15–17]) or at night [18,19]. However, desert-dwelling species and others in hot climates may enter torpor at *T*_a_ greater than 30°C [18,20]. The high *T*_a_s at which these bats begin to thermoconform approach the normal homoeothermic body temperature of most mammals (*ca* 36–38°C). In much of the lowland tropics, *T*_a_ remains high throughout the year despite large variation in rainfall and subsequent food availability, and this low variation in *T*_a_ could make measuring low energy states via *T*_b_ a challenge.

These small differences in *T*_a_ and *T*_b_ may mask that tropical mammals are more metabolically labile than we have been able to appreciate [21,22]. The estimates of energetic expenditure that skin or *T*_b_ give may not indicate reduced metabolic states [1,7], and it is increasingly apparent that *T*_b_ alone is not wholly representative of the energetic expenditure of tropical animals [7,11,18,22–25]. We need additional ways to measure how tropical mammals minimize energetic expenditure in environments with high *T*_a_, such as the tropics. One such measure would be variation in heart rate (*f*_H_) and delivery of oxygen throughout the body. Adjustment of heart rate at high *T*_a_ would allow individuals to enter low metabolic states at high *T*_b_, thus saving energy while staying alert and avoiding predation [26]. In addition, mostly due to methodological restrictions, most work has focused on measuring physiological responses to controlled variation in *T*_a_ [16] instead of under natural conditions, which might not reveal the full range of animals' physiological capacity [22,27–29].

Heart rate provides a measure of energy expenditure independent of *T*_b_ measures and can help clarify the energetic strategies used by animals in areas with low variation in *T*_a_ [30–32]. Because *f*_H_ is proportional to oxygen consumption, and therefore the MR, it is a more direct measure of instantaneous individual energy consumption [32–34]. Reduction in *f*_H_ is one of the first measurable aspects of a torpid state, with the metabolic rate falling at the same rate as *f*_H_. As *T*_b_ is a consequence of the MR, it subsequently falls at a slower rate to a controlled set point [35–37]. This lagging relationship between *f*_H_ and *T*_b_ in torpor provides the opportunity for independence of these two aspects of metabolism that can be exploited by tropical animals at high *T*_a_ [36,38]. Reduced MR and torpor at high *T*_b_ in hibernating subtropical marsupials, lemurs and arid-adapted golden spiny mice and bats [12,18,25,39,40], all indicate that *f*_H_ may be a better predictor of low energy states than *T*_b_ or the difference *T*_b_ − *T*_a_ (*T*_diff_), particularly when *T*_diff_ is small, such as in the tropics.

Our goal was to test if Pallas' mastiff bats (*Molossus molossus*) enter a state of low MR, measured through *f*_H_, even though there is little room for its *T*_b_ to be depressed in a typical torpor state. In captivity *M. molossus* modulate their body temperature from *ca* 28°C during the day (2°C higher than *T*_a_) to 34–35°C during the night when not flying [41], but there are no measures of field *T*_b_. In our field site in Gamboa, Panamá, these 10–12 g bats forage in social groups to maximize the probability of locating ephemeral insect swarms in open air. They forage for 30–40 min per night, but return to their roosts 20% heavier [42–44]. In addition to this short window of food availability, inclement weather can disperse insect clouds and prevent animals from flying. This makes *M. molossus* susceptible to unpredictable food shortages and the use of low energy states (torpor) may be particularly advantageous in these scenarios. *Molossus molossus* spend 23 h in their roost with *f*_H_ as low as 40–50 bpm and resting rates of 156 ± 71 bpm at night [43]. These resting rates are 50% lower than expected and indicate that the bats may employ torpor at high *T*_a_, and presumed high *T*_b_.

We hypothesized that *M. molossus* would thermoconform at high *T*_a_, with similar low MRs across a wide range of *T*_a_. Subsequently, low *f*_H_ will be used by *M. molossus* across a wide range of *T*_a_. We established relationships among *f*_H_, *T*_b_, *T*_a_ and energy consumption in captive individuals, and then applied these relationships to free-ranging bats roosting in their natural social groups.

## 2. Material and methods

### 2.1. Animal capture and marking

We captured 11 *M. molossus* (10.5 ± 0.7 g) with mist nets as they emerged from their roosts in holes and crevices underneath houses in Gamboa, Panamá. We marked each bat with a subcutaneous temperature-sensitive PIT-tag (BioThermo13, Biomark Inc., Boise ID, USA) [26,41] and fitted it with an external heart rate transmitter (*ca* 0.8 g; SP2000 HR Sparrow Systems, Fisher, IL USA) that emits a continuous long-wave signal, interrupted by cardiac muscle potentials [34,45]. To attach the heart rate transmitter, we trimmed the fur in the middle of the back below the shoulder blades, applied a topical analgesic (Xylocaine gel, Astra Zeneca, Wedel Germany), and disinfected the skin and electrodes with 70% EtOH [43]. We inserted the transmitter's two disinfected gold electrodes *ca* 3 mm dorsally through a puncture made with a 23 ga sterile needle. The transmitters were mounted on thin, flexible cloth and then glued over the electrode insertion points using a silicone-based skin adhesive (Sauer Hautkleber, Manfred Sauer, Germany). The electrodes were flexible and did not appear to disturb the animals. Transmitters represented 7.0 ± 0.7% (s.d.) of body mass [46]. We removed transmitters immediately after respirometry experiments or after 2–3 days of deployment on free-ranging bats. We saw no signs of infection at the lead insertion sites, and *f*_H_ and *T*_b_ records did not indicate elevation in the MR consistent with an immune challenge. Bats either maintained body mass or gained up to 1 g of mass (0.6 ± 0.3 g), revealing no measurable negative impact of the short-term deployment of the additional mass of the transmitter.

### 2.2. Laboratory measurements of heart rate, body temperature and metabolic rate

We used an open-flow, push-through respirometry system [47] to measure rates of oxygen consumption ($\dot{V}O_{2}$), carbon dioxide production ($\dot{V}{\mathrm{CO}}_{2}$) and *T*_b_ of six bats for 10–20 h continuously [48]. Owing to logistical constraints, these were not the same bats that were tracked in the field. We dried incurrent air (greater than 75% relative humidity, approx. 26°C) with Drierite (WH Hammond Drierite Co, Ltd, Xenia, OH, USA) and pumped it through a mass flow controller (FB8, Sable Systems International, Las Vegas, NV, USA) into a 1.97 l respirometry chamber fitted with a thermocouple within a 20 l insulated cooler that was dark and temperature-controlled (PELT5, Sable Systems). An additional empty chamber served as a reference (baseline) to the animal chamber. The flow rate was 300 ml min^−1^ and relative humidity and vapour production were measured with an RH-300 (Sable Systems). After drying the excurrent air again with Drierite, we measured CO_2_ concentration (FOXBOX, Sable Systems), and after scrubbing the air of CO_2_ with Ascarite (Thomas Scientific, Swedesboro NJ, USA), we determined O_2_ concentrations (FOXBOX, Sable Systems). Chamber temperature, CO_2_, O_2_ and relative humidity were recorded at 1 Hz directly with Expedata via the UI-2 data acquisition interface (Sable Systems). Bats had the option to roost on vertical or horizontal mesh platforms above a layer of mineral oil used to trap excrement. We kept the bats at 28 and 32°C for at least two hours at a time, which is equal to or lower than the lower critical threshold of the thermoneutral zone (TNZ) for this species [49]. We measured bat *T*_b_ via PIT tag (BioThermo13, Biomark, Inc.) every minute using an antenna (HPR Plus, Biomark) in the insulated chamber [41], and we recorded *f*_H_ as a sound file (see below). PIT tag calibrations against a thermometer traceable to the U.S. National Bureau of Standards showed a mean measurement error of 0.21 ± 0.2°C. *f*_H_ was averaged over the 1 min preceding each *T*_b_ measurement. This gave five *T*_b_ and *f*_H_ measures for each measurement of $\dot{\text{V}}{\text{O}}_{2}$ and $\dot{V}{\text{CO}}_{2}$. We used carbon dioxide ($\dot{V}{\mathrm{CO}}_{2}$) production to estimate metabolic rates using Equation 10.5 from Lighton [50]: $\dot{V}C{\text{O}}_{2}=({\text{FeCO}}_{2}{\text{-FiCO}}_{2})*\mathrm{FR}/(1{\text{-FeCO}}_{2}*(1\text{-}(1\text{-RER})))$, where FiCO_2_ is the incurrent CO_2_ content, FeCO_2_ is the excurrent CO_2_ content, FR is the flow rate and RER is the respiratory exchange ratio ($\dot{V}C{\text{O}}_{2}$:$\dot{V}{\text{O}}_{2}$), which we calculated to be 0.8 from empirical measurements of CO_2_ and O_2_. We converted $\dot{V}C{\text{O}}_{2}$ (ml min^−1^) to metabolic rates [51,52] that would be comparable to field rates using the standard conversion of 25.0 J ml^−1^ CO_2_. After conclusion of the experiment, the heart rate transmitters were removed, bats were offered water via a transfer pipette and placed in the entrance to their roost.

### 2.3. Field heart rate and body temperature telemetry

We recorded *f*_H_ and *T*_b_ of five bats that were not part of the respirometry experiments during 1–3 days and nights in their natural roosts. We used telemetry receivers (AR8000, AOR Ltd) connected to 3-element Yagi antennae (Sparrow Systems) to detect the signal of the heart rate transmitter. This was then recorded via the mini-dv output to a wave file on a digital recorder (Tascam DR-05). The maximum recording distance for *T*_b_ was 10 cm, and 100 m for *f*_H_ transmitters; therefore, no flying data were recorded. Three bats (1646, 1721, 1732) roosted in the walls of a wooden house and had both *f*_H_ and *T*_b_ sampled every 10 min. The heart rate was recorded continuously for two bats 2253 and 2289, and *T*_b_ was recorded once per minute. Bat 2253 roosted in the roof of a wooden structure under a metal roof and bat 2289 in the brick ground floor of a house. To temporally synchronize *T*_b_ and *f*_H_, a smoothed *f*_H_ of the previous 60 s was matched to each *T*_b_. Ambient temperature data were recorded at 15 min intervals by the Autoridad del Canal de Panamá (ACP) for Gamboa and provided by the Smithsonian Tropical Research Institute's Physical Monitoring Program. The daytime mean ambient temperature was 25.87 ± 1.21°C (mean daytime minimum to mean maximum: 23.38–28.24), and the mean nightly ambient temperature was 23.74 ± 0.50°C (mean nightly minimum to mean maximum: 22.74–24.78°C). The ACP monitoring station was located along the banks of the Panama Canal 400–800 m from the observation roosts. While these temperatures do not directly measure the more insulated and stable microhabitats the bats experience in their roosts, they show the potential *T*_a_ that bats experience across the day.

### 2.4. Heart rate scoring

The heart rate from radio transmitters has been visually scored at sampling intervals of 5–10 min [33,34,45,53]. However, complete sampling can show novel energy-saving strategies like those in tent-making bats that depress *f*_H_ several times per hour [26], a pattern that would not have been detected by sampling every five to ten minutes. We therefore fully sampled the recorded data using an automated approach in R 3.3.2 [54] to identify and count heartbeats [26]. We applied a finite impulse response filter in *seewave* [55] with a window length of 1500–2000 samples to select the carrier frequency of the transmitter. We counted individual heartbeats by applying a timer function in *seewave* that ran over non-overlapping windows of 500 samples. This created a resolution of 88–96 sampling windows per second. We then applied a kernel density filter in *KernSmooth* [56] to further eliminate noise that was outside of the 90% quantile. This approach is conservative and may have eliminated some heart rate outliers, but the autocorrelated nature of heart rate allowed us to filter out errors probably induced by static or other interference in the recordings. Automated samples were visually inspected periodically to validate the filtering method, particularly when there was high variation.

We could not apply all the automated methods to the respirometry data due to large amounts of interference from the PIT tag reader within the respirometry chamber. Here, we hand-scored *f*_H_ by counting all heartbeats within the first 10 s of every minute. Sampling at regular intervals may underestimate short-term changes in *f*_H_ [26], but the consistent low *f*_H_ of the bats throughout our captive and field experiments did not warrant finer-scale sampling. This was the same time resolution as *T*_b_ measures and provided five *f*_H_ measures per unit of MR sampling. We then averaged these measures to create a single value for each 5 min respirometry sample.

### 2.5. Analysis

We tested the fit of heart rate (*f*_H_), body temperature (*T*_b_), and the difference between body temperature and ambient temperature (*T*_diff_ = *T*_b_ − *T*_a_) on the metabolic rate (MR) using generalized linear mixed-effects models (GLMMs) with individual as a random intercept in *lme4* after inspecting the data for normality and equal variance. We used both the field metabolic rate (FMR, kJ h^−1^) and mass-specific metabolic rates (W g^−1^) to facilitate broader comparisons with published data, particularly those collected in field experiments. We compared the minimum metabolic rates of *M. molossus*, identified as the lowest 10% quantile of the MR for each individual, to minimum torpor metabolic rates for species found across a variety of temperature regimes (temperate, subtropical and tropical) that undergo daily torpor from three speciose mammalian orders (Chiroptera, Dasyuromorphia and Rodentia; data from Ruf and Geiser [13]). A nested model approach revealed that all three of our predictors (*f*_H_, *T*_b_, *T*_diff_) contributed significantly to explaining the variation in both measures of energy consumption (FMR and mass-specific MR). We therefore took a model selection approach to evaluate which factors were the most efficient at predicting energy consumption in respirometry. We calculated the Akaike information criterion corrected for small sample sizes (AICc) for each model, as well as the confidence intervals for each model parameter in *lme4*, and both the marginal ($R^{2}{}_{m}$, fixed effects alone) and conditional ($R^{2}{}_{c}$, full model) *R*^2^ values in *MuMIn* [57] using the approach outlined by Nakagawa & Schielzeth [58]. All analyses were performed in R 3.3.2 [54], and we present means ± s.d. for all variables unless otherwise noted.

## 3. Results

### 3.1. Respirometry, captive heart rate, body temperature and energy consumption

**Figure 1.**
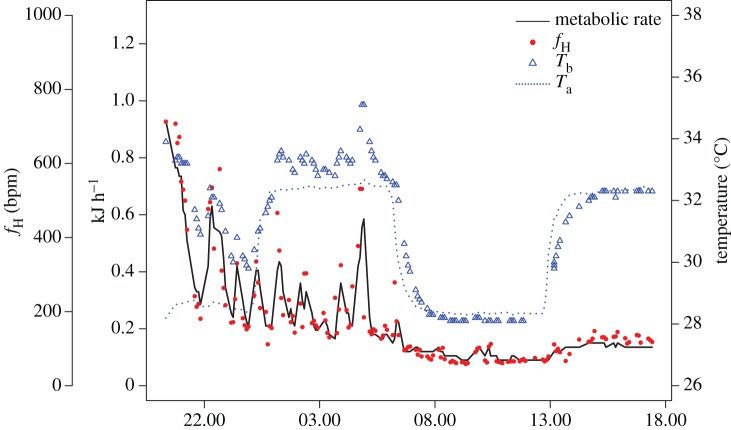
Metabolic rate (black line), *f*_H_ (red circles), *T*_b_ (blue triangles) and *T*_a_ (dotted line) of an exemplary *M. molossus* measured across 20 h in open-flow respirometry.

**Figure 2.**
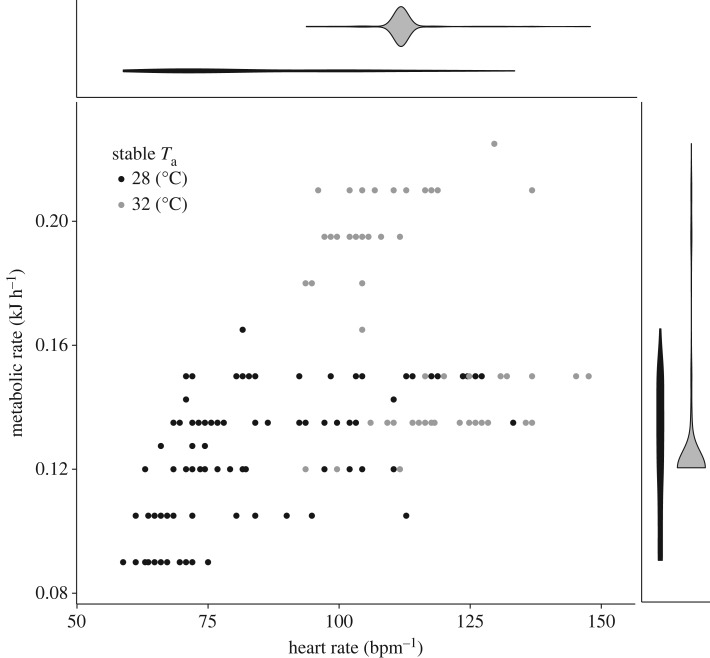
Metabolic rate (kJ h^−1^) and heart rate (bpm) of bats during steady-state minimum measures at *T*_a_ of 28 and 32°C during respirometry experiments. Violin plots show the distribution and density of heart rate (top) and metabolic rate (right) at each temperature. Note that all heart rates remained below 150 bpm.

All bats reduced their MR and *T*_b_ in the respirometry chamber. We recorded a large range of MR (0.075–1.245 kJ h^−1^ or 0.0021–0.325 W g^−1^), *f*_H_ (59–999 bpm) and *T*_b_ (27.9–37.6°C) across the 10–20 h of continuous sampling. In general, *T*_b_ followed *T*_a_ when the MR was reduced across the small range of *T*_a_ that we measured, and *f*_H_ was not dependent on *T*_b_ (figure 1 and electronic supplementary material, figure S1). After physiological arousal generated by observers tapping on chamber walls, *f*_H_ dropped rapidly into a low metabolic state, and *T*_b_ eventually followed at a slower rate. Bats showed a low MR of 0.1218 ± 0.021 (mean ± s.d.) kJ h^−1^ (0.00314 ± 0.00051 W g^−1^). When we further examined the stable MR at our two *T*_a_, we found that metabolic rates were lower at 28°C (0.131 ± 0.014 s.e. kJ h^−1^) than at 32°C (0.160 ± 0.001 s.e. kJ h^−1^; $\chi_{1}^{2}=445.77$ , *p* < 0.001; figure 2). These lower MR at 28°C are consistent with lower *f*_H_ at 28°C (82.4 ± 6.3 bpm) than at 32°C (115.8 ± 1.0 bpm; $\chi_{1}^{2}=1059.2$, *p* < 0.001; figure 2).

**Figure 3.**
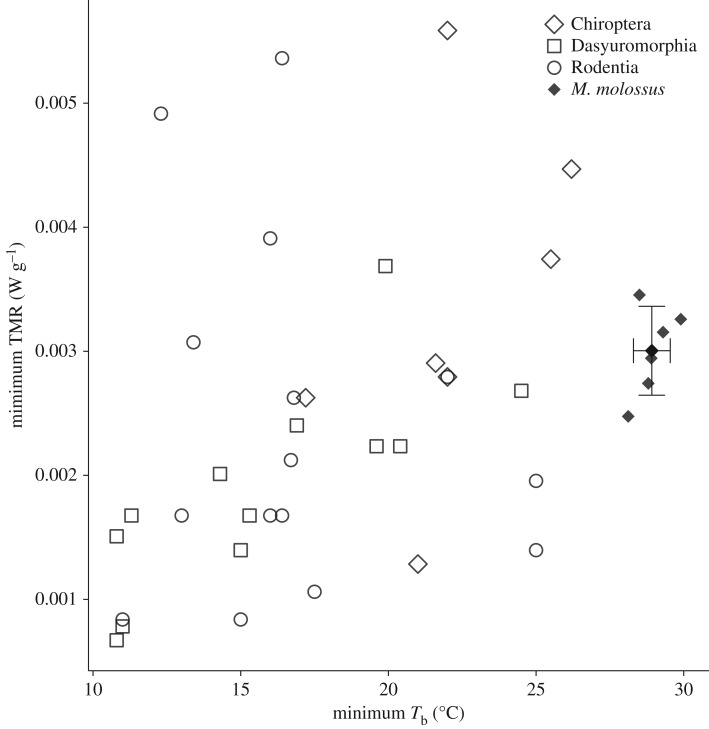
Minimum torpor metabolic rates (TMRs) and minimum *T*_b_ for Chiroptera, Dasyuromorphia and Rodentia that use daily heterothermy (adapted from Ruf & Geiser [13]), and the mean 10% quantile values (± s.d.) for individual *M. molossus. Molossus molossus* use the same low range of torpor metabolic rates at higher *T*_b_ as other mammals that use daily heterothermy.

*Molossus molossus* generally used low mass-specific MR in the respirometry chamber. Bats showed a low minimum MR of 0.1138 ± 0.0167 kJ h^−1^ (0.00294 ± 0.00034 W g^−1^) and this did not differ from the minimum metabolic rates for other mammalian orders that use daily heterothermy ($\chi_{3}^{2}=5.525$, *p* = 0.154; figure 3). *Molossus molossus* enter low metabolic states at substantially higher *T*_b_ than these other mammals ($\chi_{3}^{2}=26.68$, *p* < 0.001; figure 3).

**Figure 4.**
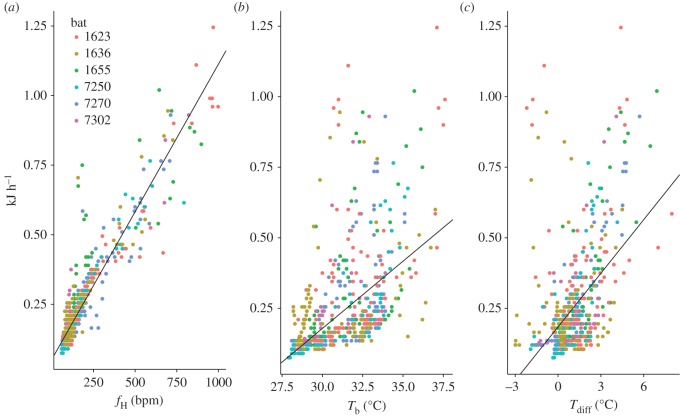
The relationship between *M. molossus* metabolic rate (kJ h^−1^) and (*a*) *f*_H_, (*b*) *T*_b_ and (*c*) *T*_diff_. In all models evaluated, *f*_H_ has the best predictive fit for energy consumption (electronic supplementary material, table S1).

All three predictor variables (*f*_H_, *T*_b_, *T*_diff_) explained substantial variation in the MR, but *f*_H_ alone was the best model regardless of any temperature interaction (figure 4; electronic supplementary material, table S1). All models that included *f*_H_ had low AICc values and high *R*^2^. Models including either *T*_b_ or *T*_diff_ resulted in better fits, but the increase in model fit provided by adding in a temperature measure came with a high penalty of AICc. The wide range of *T*_b_ at any given MR and between the two temperature regimes illustrates the low predictive ability of both *T*_b_ and *T*_diff_ (figures 2 and 4). The best-fit model predicted energetic expenditure as daily energetic expenditure (kJ d^−1^) = 0.00106*f*_H_ + 0.0527 ($R^{2}{}_{c}=0.88$). *T*_a_ had minor effects on *f*_H_, showing a small but significant increase in *f*_H_ with rising *T*_a_ ($\chi_{1}^{2}=51$, *p* < 0.001; *f*_H_ = 7.41 × *T*_a _− 115.67; $R^{2}{}_{c}=0.22$; electronic supplementary material, figure S2 and table S2). There was a stronger relationship between *T*_b_ and *f*_H_ ($\chi_{1}^{2}=398$, *p* < 0.001; *f*_H_ = 14.985 × *T*_b_ − 353.237; $R^{2}{}_{c}=0.55$; electronic supplementary material, table S2).

### 3.2. Free-ranging heart rate and body-temperature telemetry

**Figure 5.**
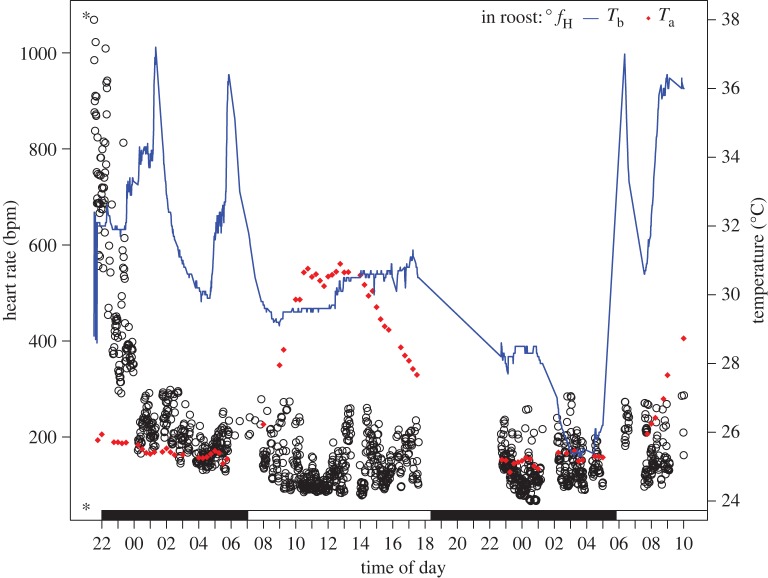
In-roost *f*_H_ (open circles), *T*_b_ (blue line) and *T*_a_ (red diamonds) measured for bat 2289 across a 36 h period. The asterisk (*) indicates where the bat was released back to its roost. Missing *f*_H_ and *T*_b_ data coincide with the bat's foraging period and equipment adjustment. Scotophase is indicated by the filled bar along the bottom.

The mean *f*_H_ of free-ranging *M. molossus* in their roosts were generally low across the 24 h period but were in the range of 58–1068 bpm. Heart rate varied most in the early evening period when bats returned from foraging or were returned to their roost after instrumentation (figure 5). The heart rate was elevated to over 1000 bpm during those times. Mean roosting *f*_H_ were below 200 bpm for all periods of the day and night (147 ± 57 bpm) and remained in stable low-level states (electronic supplementary material, figure S3).

**Figure 6.**
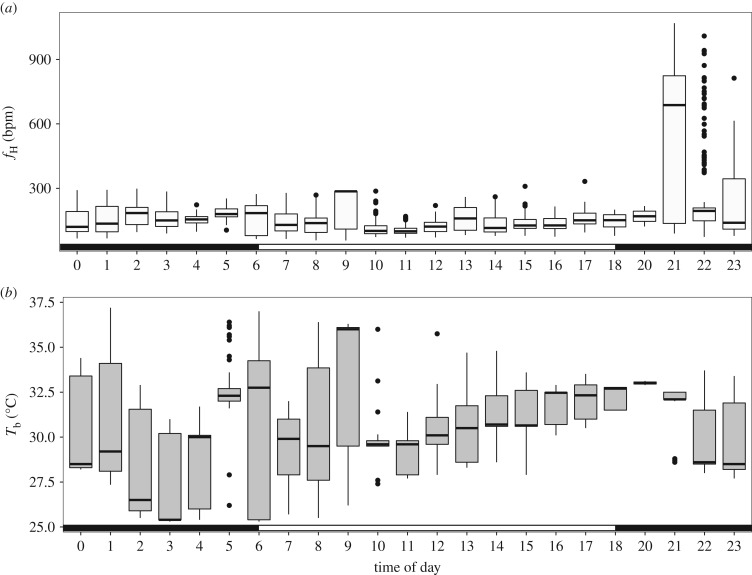
Hourly means of *f*_H_ (*a*) and *T*_b_ (*b*) simultaneously measured from bats in their natural roosts. Scotophase is indicated by the filled bar along the bottom of each panel.

*T*_b_ of roosting bats were in the range of 25.3–37.2°C and varied both within an hour and across the day (figure 6). *T*_b_ increased with daily *T*_a_ ($\chi_{1}^{2}=39.7$, *p* < 0.001; slope = 0.481, ${R^{2}}_{m}=0.121,\;{R^{2}}_{c}=0.461$). *f*_H_ in the lowest 10% of observed values (i.e. lower than 90 bpm) were observed in all hours of the day except 2, 5, 19 and 20 h. These low rates were observed at *T*_b_ up to 33°C, and *f*_H_ of 100 bpm was observed at up to 36°C (electronic supplementary material, figure S3).

Our in-roost *T*_b_ measures indicated that *M. molossus* used heterothermy, with minimum *T*_b_ occurring during 02.00–04.00 in the coolest portion of the night. As daily *T*_a_ increased during the diurnal period, *T*_b_ became less variable, with individuals maintaining a constant *T*_b_ that was close to *T*_a_. When individual variation was accounted for, *T*_b_ was mildly related to *f*_H_ ($\chi_{1}^{2}=652.0$, *p* < 0.001; slope = 10.12 ± 0.37 (s.e.m.); ${R^{2}}_{m}=0.215,\;{R^{2}}_{c}=0.450$; electronic supplementary material, figure S3). The *T*_a_ and *T*_b_ differential was also related to *f*_H_ ($\chi_{1}^{2}=44.15$, *p* < 0.001; slope = 6.97 ± 1.05, ${R^{2}}_{m}=0.151,\;{R^{2}}_{c}=0.426$) across a range of *T*_diff_ (*f*_H_ ∼ *T*_b_ AIC = 21 720, *N* = 2210; *f*_H_ ∼ *T*_diff_ AIC = 2230, *N* = 224; electronic supplementary material, table S2 and figure S3).

*T*_diff_ explained 34% of the variation in *f*_H_ when inter-individual variation was accounted for via random effects in our models. This is best illustrated by individual daily variation in these variables (figure 5). In the example we illustrate, the bat roosted between a wooden ceiling and a metal roof approximately 2 m above the ground. We could not measure the interior temperature of the roost, but *T*_b_ measured through the PIT tag reader ranged from 25.3 to 37.2°C and heart rate was generally low.

## 4. Discussion

We found that *Molossus molossus* entered low energy states independent of *T*_a_, which results in substantial energy savings across much of the *T*_a_ they experienced. In respirometry chambers, *f*_H_ was the best predictor of metabolic rate. These results extended to free-ranging *M. molossus* that used low *f*_H_ across nearly the full range of *T*_b_ measured. Minimizing energy expenditure in tropical settings with low variation in high *T*_a_ may then be possible through modifications of heart rate and oxygen consumption independent of *T*_b_. Such physiological adaptations are perhaps vital for tropical lowland endotherms because they free them from some of the energetic constraints of high *T*_a_, but these effects remain understudied like many aspects of tropical ecosystems [59].

A range of mammals achieve low metabolic rates at relatively high *T*_b_, described as potential ‘hyperthermic daily torpor’ by Lovegrove *et al.* [8]. For example, in gerbils (32–35°C) [40], fat-tailed dwarf lemurs (30°C) [25] and some desert dwelling bats (31–33°C, [18,19]), *T*_b_ thermoconforms at high *T*_a_ while the animals remain in low metabolic states, and the MR then increases with rising *T*_a_. Bats appear to be particularly flexible in their thermal profiles, and some are even able to thermoconform to *T*_a_ > 45°C for extended periods of time, which would be lethal in many other mammals [19,60]. This may be related to the high *T*_b_ generated during flight [14], or to the high TNZ upper critical temperature (e.g. 38°C) found in many species [49], although the thermoneutral dynamics for most bat species are underexplored. In previous work on tropical and subtropical bats, the finding of a reduction in *T*_b_ associated with heterothermy is variable, and they only show heterothermy when *T*_a_ is below 20–25°C [29,61–64]. However, many of these species, particularly those studied in free-ranging field conditions, have only had *T*_b_ measured. It is possible that like *M. molossus*, they enter low energy states at relatively high *T*_b_ and maintain low heart rates across a wide range of *T*_a_ and *T*_b_. Entering a low energy state at high *T*_b_, or a *T*_b_ within the thermoneutral zone, would allow animals to lower their FMR without the costs of rewarming that are incurred as they leave torpid states [65], while taking advantage of the benefits of heterothermy with minimal costs [7,8]. Passive warming would be particularly advantageous for nocturnal animals that enter their active periods as *T*_a_ lowers and is less likely to provide much passive thermal support in their transition towards active states.

Our respirometry measures did not capture the full metabolic potential of *M. molossus* observed in the wild roosts*.* The *T*_a_ that we used in our respirometry measures did not extend far beyond the lower critical temperature (*ca* 30°C) of the thermoneutral zone reported for *M. molossus* [49], but energy consumption at these temperatures was far lower than the basal metabolic rate (BMR) reported for this species, and steady-state MRs were lower at 28°C than at 32°C, with a mean difference of 0.029 kJ h^−1^. The mean of the 30% quantile of respirometry MR (0.00314 ± 0.0005 W g^−1^) was only 39% of the previously measured BMR for *M. molossus* (0.008044 W g^−1^ [49]). We did not observe this value until the 90% quantile of our data, placing it among the highest MRs we recorded for our bats. The high temporal variability in metabolic rate indicates that bats did not employ a stable BMR in our respirometry measurements, despite extended measurement periods and *T*_a_ that should be within a neutral range for this species. Accurately measuring the BMR and the thermoneutral zone is difficult when species thermoconform across a wide range of *T*_a_ [7,66]. Our data show that measuring this type of energetic and thermal flexibility is important, and that we may find that this energetic flexibility is common in tropical bats.

Heart rate is an accurate measure of the metabolic rate during steady-state conditions and when transitioning between resting states. However, torpor with strong heterothermy is not just an extrapolation of a resting state as regressions of torpid bats would underestimate resting $\dot{V}{\text{O}}_{2}$ by up to 75% [67]. The low metabolic states in *M. molossus* do not show such a curvilinear relationship, with linear models providing the best fit to our respirometry data. We are careful not to infer a continuous linear relationship once animals are exercising because this probably underestimates the metabolic rate during exercise [30]. Through careful calibration and the use of nonlinear exponential estimates of energy consumption from *f*_H_ based on body mass and heart mass, it may be possible to even more accurately estimate the energetic expenditure of exercising free-ranging animals.

The regular low metabolic states used by *M. molossus* in both respirometry and natural roosts may be an overall energetic conservation strategy to compensate for their ephemeral food resources. These small bats typically forage over water bodies for less than an hour per night on unpredictable, but rich patches of insects at dusk and dawn [42]. When nights are particularly windy or rainy, these bats tend to forgo foraging (DKN Dechmann, unpublished data), and there is a positive relationship between the duration of foraging and *f*_H_ in the roost [43]. Furthermore, we observed *T*_b_ reductions across a wide range of *T*_a_, with free-ranging bats using *T*_b_ of 25.3–37.2°C. Multi-day torpor bouts have not been observed in *M. molossus* and we did not attempt to measure this in our experiment. While *M. molossus* are capable of maintaining stable blood glucose levels for up to 48 h of fasting [68], total reduction of the MR through extended torpor-like states may allow them to cope with multiple nights of inclement weather. In some cases, *T*_b_ was lower than the *T*_a_ recorded at the Panama Canal (electronic supplementary material, figure S2). Bats then select cool, stable roost microhabitats regardless of their ability to maintain a low MR at high *T*_b_. The use of torpor by tropical and subtropical mammals [13,16,22,25,69,70] illustrates the utility of reductions in metabolic rates and *T*_b_ during periods of low food availability. However, starvation risk is not the only driver of torpor, as well-fed bats will also use torpor to minimize time outside the roost [17]. These torpor and torpor-like states at high *T*_b_ may be particularly important to reduce the evaporative water loss incurred by bats via the large naked membrane of their wings [14], which can be reduced by 90% during torpid states [71,72]. The possibility of low energy states at relatively high *T*_b_ allows *M. molossus* to remain active and alert and move away from observers. This means that unlike the lethargic torpid bats with low *T*_b_ in the temperate zone, bats at higher *T*_b_ can escape from predators at any given time [71,73]. Daily torpor-like states in *M. molossus* allow them to minimize exposure to risks outside of their roost (such as predators and water loss), while maximizing their energy savings.

## 5. Conclusion

We suggest that the low energy state that we measure in *M. molossus* is torpor with shallow heterothermy. In other small mammals, minimum *f*_H_ during daily torpor at low *T*_b_ is near 70 bpm [36,37,74–76]. This is similar to the minimum stable rates (58–75 bpm) of *M. molossus* both during respirometry and in their natural roosts, but much higher than the *f*_H_ of 8 bpm for hibernating bats [67]. The metabolic rates used by our bats were well within the ranges reported for other mammalian orders that use daily heterothermy (figure 3), but occur at higher *T*_b_. Bats evolved in the tropics and our findings in *M. molossus* are probably an example of the basal form of heterothermy that evolved near the root of the mammalian radiation [77]. The ability to maintain low metabolic rates and subsequently low *T*_b_ in deep torpor or hibernation would build upon this set of regulatory networks driven by cellular requirements for oxygen diffusion via *f*_H_, which reduced total metabolic rates while in thermoneutral conditions or above. Tropical heterothermy or torpor may allow individuals to flexibly adjust energetic expenditure to rapid environmental changes at fine timescales, as well as minimize energetic expenditure during pregnancy and lactation when *T*_b_ reductions may be constrained [7,17,64,78].

Low *f*_H_ in response to lowered oxygen demands is an effective, flexible strategy. Work integrating energetic expenditure via *f*_H_ in birds repeatedly shows lower energetic expenditures than would be predicted [33,34,45,79,80], indicating that a diversity of adaptations may be possible by manipulating one of the primary drivers of energy delivery to metabolism. The widespread nature of heterothermy or torpor-like states in tropical species leads to challenges when using traditional cut-off *T*_b_ or frequency distributions of *T*_b_. Heart rate studies in tropical bats, including this study, have shown surprising metabolic strategies to cope with life in warm ambient temperatures [21,26]. This direct information on the flow of energy through an individual allows us further insight into the variability of energetic strategies in tropical systems, particularly as ambient conditions become more unpredictable.

## Supplementary Material

ESM Figure S1. Respirometry measures of Molossus molossus

## Supplementary Material

ESM Figure S2. Respirometry relationships between heart rate and temperature measures

## Supplementary Material

Figure S3. In-roost relationships between heart rate and temperature measures

## Supplementary Material

ESM Table S1. Model selection

## Supplementary Material

Table S2. Linear relationships between heart rate and body temperature

## Data accessibility

Data are available for download from Dryad http://dx.doi.org/10.5061/dryad.6620j [81].
