# Supplementary figures and images for: Heart rate reveals torpor at high body temperatures in lowland tropical free-tailed bats
Source: R Soc Open Sci. 2017 Dec 20;4(12):171359. doi: 10.1098/rsos.171359 (PMC5750026; doi:10.1098/rsos.171359)

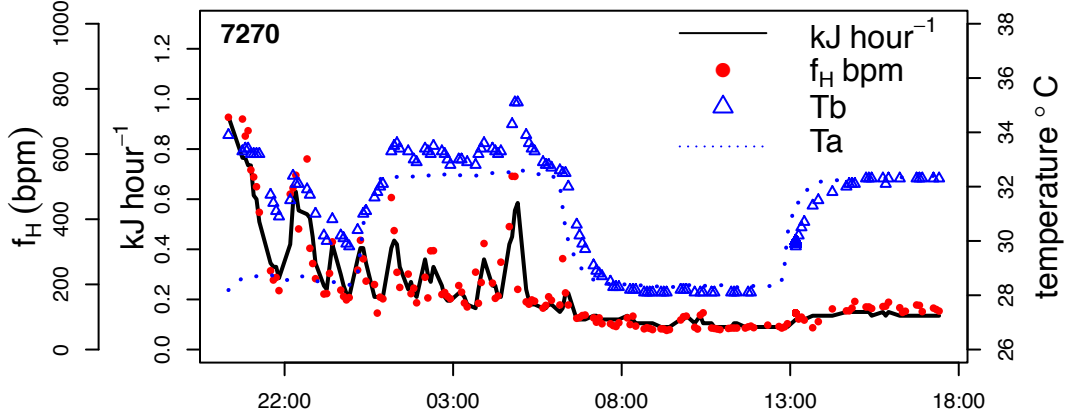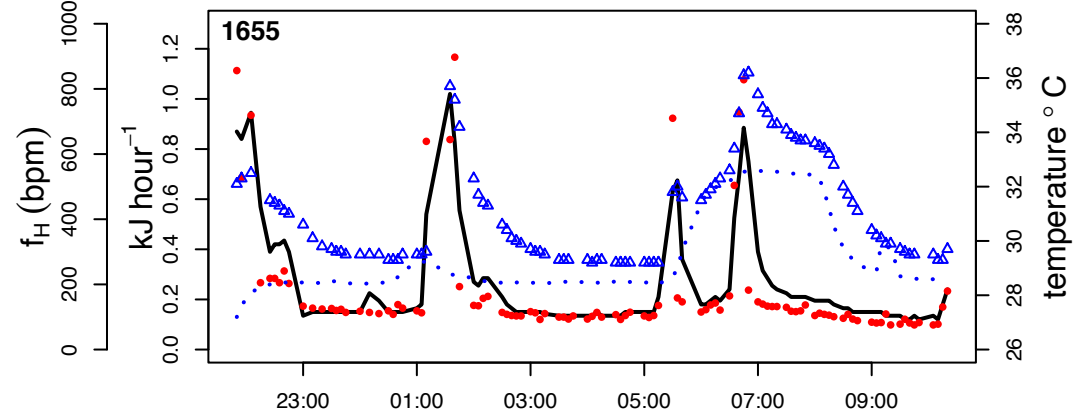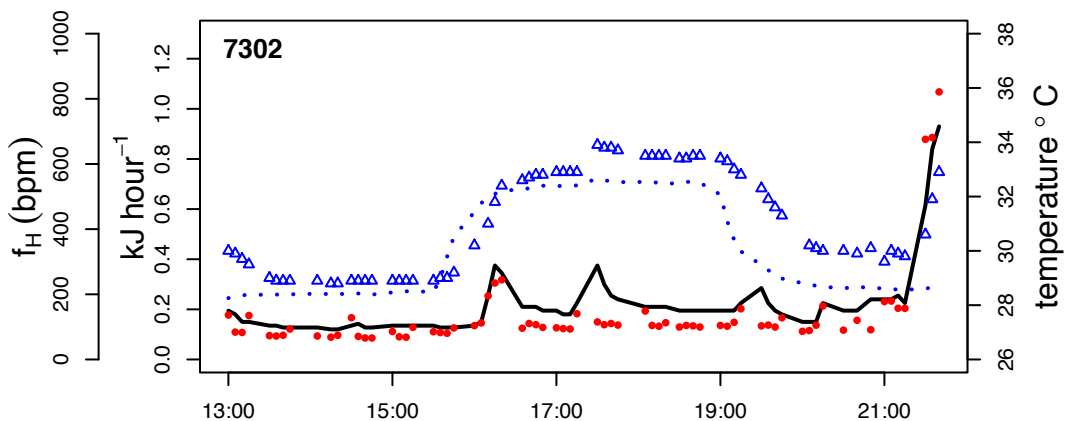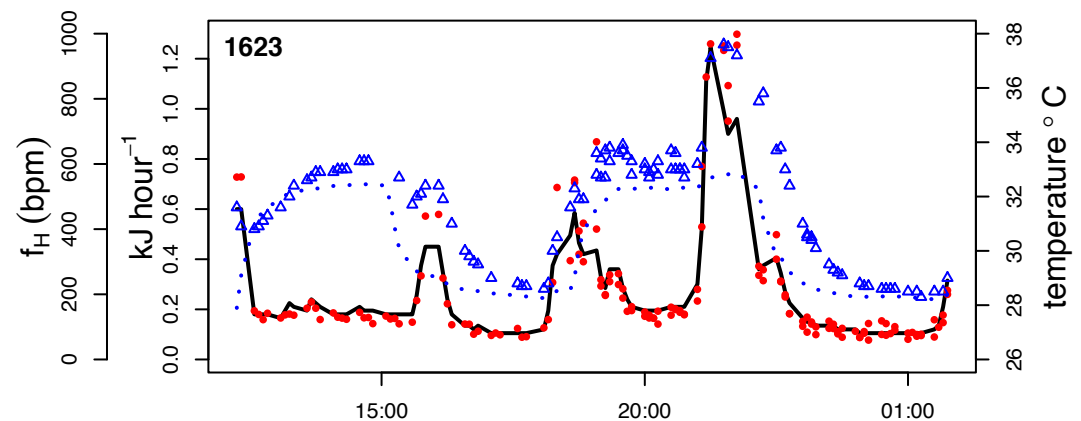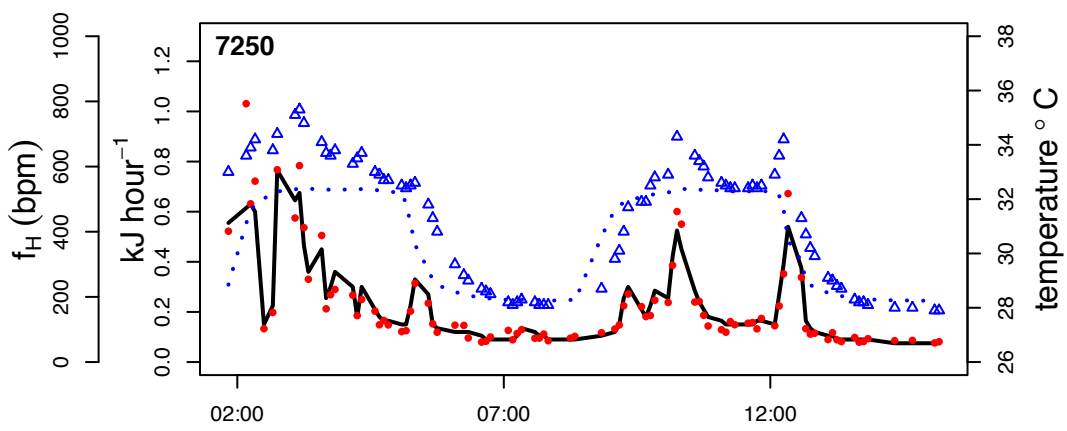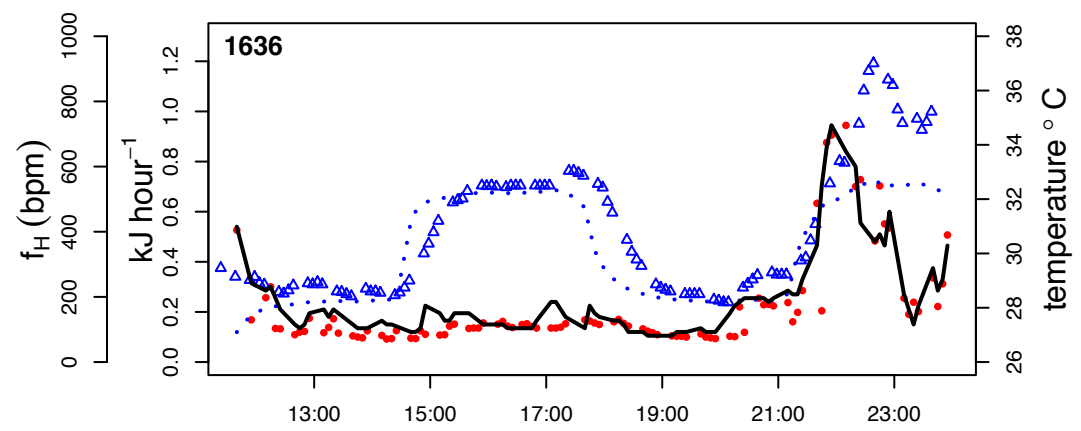

Supplement: ESM Figure S1. Respirometry measures of Molossus molossus [file rsos171359supp1.pdf]

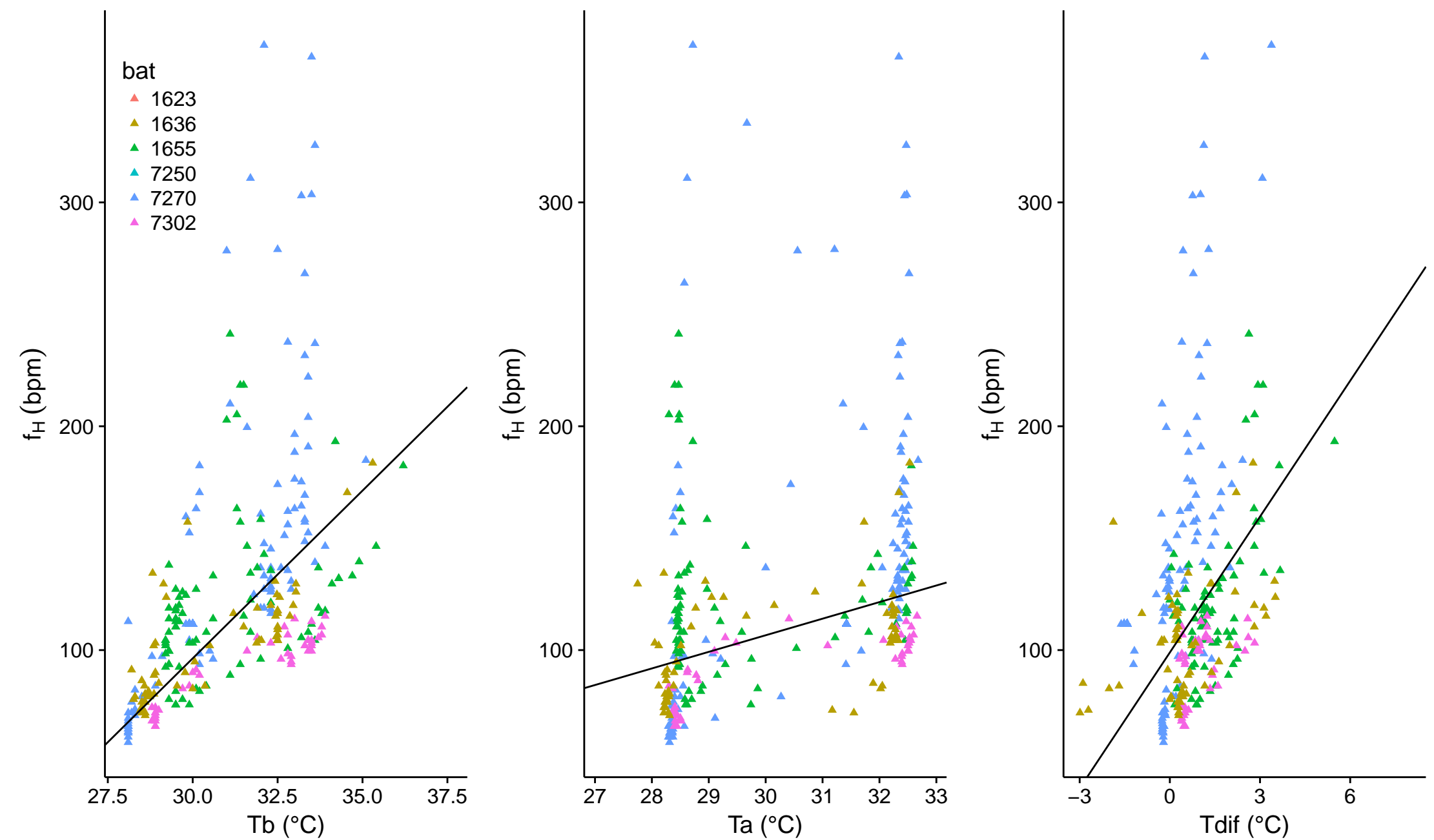

Supplement: ESM Figure S2. Respirometry relationships between heart rate and temperature measures [file rsos171359supp2.pdf]

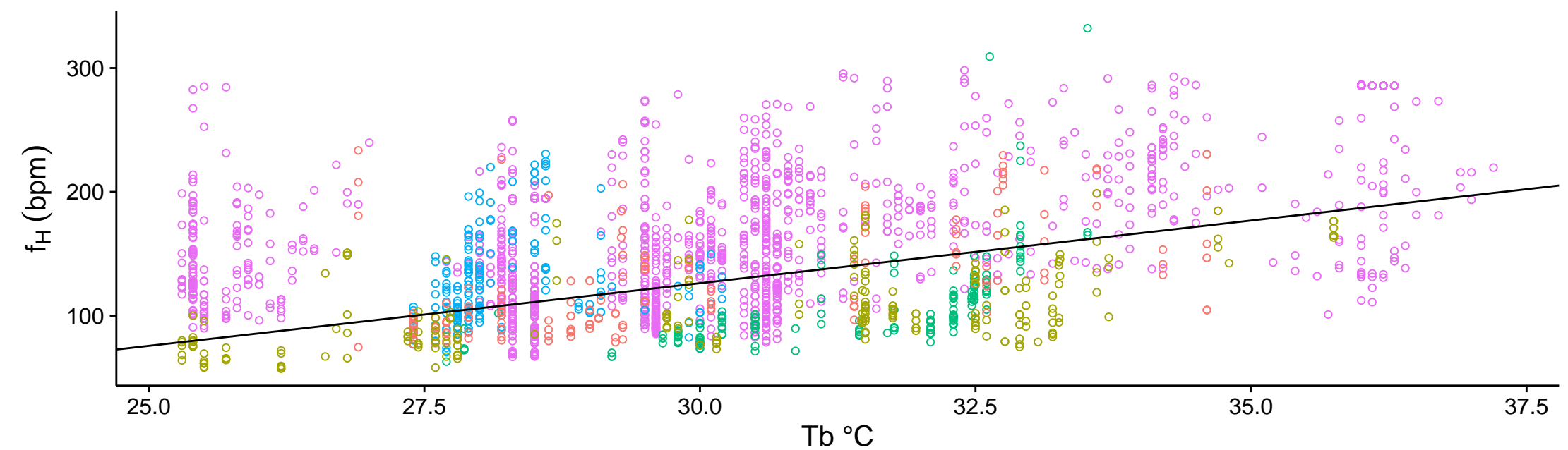

bat   1646   1721   1732   2253   2289

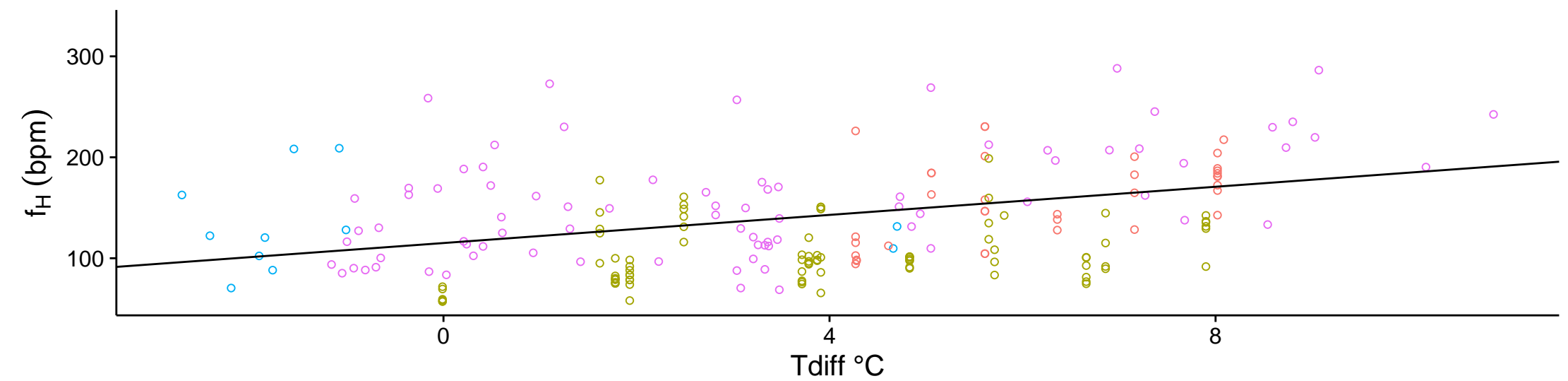

Supplement: Figure S3. In-roost relationships between heart rate and temperature measures [file rsos171359supp3.pdf]
