# Supplementary material for: Heart rate reveals torpor at high body temperatures in lowland tropical free-tailed bats
Source: R Soc Open Sci. 2017 Dec 20;4(12):171359. doi: 10.1098/rsos.171359 (PMC5750026; doi:10.1098/rsos.171359)
Supplement: ESM Table S1. Model selection [file rsos171359supp4.docx]

Table S1. GLMM model selection to estimate energy consumption via heart rate (f_H_), body temperature (T_b_) or the difference between T_b_ and T_a_ (T_diff_) in *M. molossus*. Shown are the corrected AIC value (AICc), and the model R^2^ for fixed factors only (R^2^m) and conditional R^2^ with random effects included (R^2^c).

| **Model**  **(kJ hour^-1^ ~ )** | **AICc** | **R^2^m** | **R^2^c** | **Parameter** | **Estimate** | **95% CI**  **minimum**  **maximum** |
| --- | --- | --- | --- | --- | --- | --- |
| f_H_ | -3085 | 0.86 | 0.88 | Intercept | 0.0527 | 0.03255  0.072978 |
|  |  |  |  | f_H_ | 0.00106 | 0.001032  0.001081 |
| T_b_ | -1244 | 0.30 | 0.34 | Intercept | -1.25248 | -1.39614  -1.10882 |
|  |  |  |  | T_b_ | 0.04770 | 0.04314  0.05225 |
| T_diff_ | -1264 | 0.40 | 0.41 | Intercept | 0.18410 | 0.16659  0.20161 |
|  |  |  |  | Slope | 0.06335 | 0.05755  0.06915 |
| f_H_ * T_b_ | -3021 | 0.87 | 0.90 | Intercept | -0.324 | -0.4086  -0.2970 |
|  |  |  |  | f_H_ | 0.00143 | 0.001105  0.001765 |
|  |  |  |  | T_b_ | 0.0124 | 0.009736  0.015070 |
|  |  |  |  | f_H_:T_b_ | -0.0000135 | -0.00002344  0.000003508 |
| f_H_ + T_b_ | -3038 | 0.87 | 0.89 | Intercept | -0.252 | -0.3176366  -0.186248 |
|  |  |  |  | f_H_ | 0.000990 | 0.0009634  0.001017 |
|  |  |  |  | T_b_ | 0.0102 | 0.0080634  0.012249 |
| f_H_ * T_diff_ | -3053 | 0.89 | 0.90 | Intercept | 0.0466 | 0.03368  0.05943 |
|  |  |  |  | f_H_ | 0.00103 | 0.0009945  0.001061 |
|  |  |  |  | T_diff_ | 0.0212 | 0.01737  0.02511 |
|  |  |  |  | f_H_:T_diff_ | -0.0000236 | -0.00003405  -0.00001321 |
| f_H_ + T_diff_ | -1373 | 0.4498 | 0.4548 | Intercept | 0.0534 | 0.0397899  0.06694 |
|  |  |  |  | f_H_ | 0.000983 | 0.0009563  0.00101 |
|  |  |  |  | T_diff_ | 0.0150 | 0.0121971  0.01770 |
