# Supplementary material for: Heart rate reveals torpor at high body temperatures in lowland tropical free-tailed bats
Source: R Soc Open Sci. 2017 Dec 20;4(12):171359. doi: 10.1098/rsos.171359 (PMC5750026; doi:10.1098/rsos.171359)
Supplement: Table S2. Linear relationships between heart rate and body temperature [file rsos171359supp5.docx]

Table S2. Heart rate (f­_H­_) and body temperature (T­_b_) of *M. molossus* in respirometry and of free-ranging individuals in their roosts.

| **Model**  **(f_H_ ~ )** | **AICc** | **R^2^m** | **R^2^c** | **Parameter** | **Estimate** | **95% CI**  **minimum**  **maximum** |
| --- | --- | --- | --- | --- | --- | --- |
| T_b_ |  |  |  |  |  |  |
| Respirometry | 6098 | 0.30 | 0.55 | Intercept | -353.237 | -401.618  -304.34 |
|  |  |  |  | T_b_ | 14.985 | 13.496  16.45 |
| In-roost | 21720 | 0.21 | 0.45 | Intercept | -177.491 | -211.037  -144.17 |
|  |  |  |  | T_b_ | 10.123 | 9.403  10.84 |
| T_diff_ |  |  |  |  |  |  |
| Respirometery | 6160 | 0.32 | 0.68 | Intercept | 98.93 | 70.31  127.6 |
|  |  |  |  | T_diff_ | 20.23 | 17.95  22.5 |
| In-roost | 2230 | 0.15 | 0.43 | Intercept | 115.0 | 86.561  143.549 |
|  |  |  |  | T_diff_ | 6.97 | 4.914  9.025 |
